# Different Formation Routes of Pore Structure in Aluminum Powder Metallurgy Alloy

**DOI:** 10.3390/ma12223724

**Published:** 2019-11-11

**Authors:** Jana Bidulská, Róbert Bidulský, Marco Actis Grande, Tibor Kvačkaj

**Affiliations:** 1 Faculty of Materials, Metallurgy and Recycling, Institute of Materials and Quality Engineering, Department of Plastic Deformation and Simulation Processes, Technical University of Kosice, Vysokoskolska 4, 04200 Kosice, Slovakia; tibor.kvackaj@tuke.sk; 2 Agency for the Support of Regional Development Kosice, Kosice Self-Governing Region, Strojarenská 3, 04001 Kosice, Slovakia; robert.bidulsky@arr.sk; 3 Department of applied science and technology (DISAT), Politecnico di Torino, Viale T. Michel 5, 15121 Alessandria, Italy; marco.actis@polito.it

**Keywords:** powder metallurgy, aluminum alloys, porosity, press-and-sinter, ECAP

## Abstract

In powder metallurgy (PM), severe plastic deformation (SPD) is a well-known technological solution to achieve interesting properties. However, the occurrence of pores in the final product may limit these properties. Also, for a given type of microstructure, the stereometric parameters of the pore structures, such as shape (represented by Aspect and Dcircle) and distribution (fshape, and fcircle), decisively affect the final properties. The influence of different processing routes (pressing, sintering and equal channel angular pressing (ECAP)) on pore structures in an aluminum PM alloy is discussed. The nature of porosity, porosity evolution and its behavior is explored. The correlation between pore size and morphology is also considered. The final pore structure parameters (Aspect, Dcircle, fshape, and fcircle) of studied aluminum alloys produced by different processing routes depends on the different formation routes.

## 1. Introduction

Modern processing of lightweight metals made from powders enables the development of materials with high tensile strength coupled to adequate plasticity and weight ratio with improved tolerances. Aerospace and automotive markets, in particular, need a cost-effective production of relatively complex components with the aim of fuel-saving through weight reduction of the parts. One critical factor throughout the powder metallurgy (PM) cycle may be the presence of residual porosity. Considering mechanical properties, residual porosity has a major impact on the strength of the part. 

Porosity roughly represents the fraction of void volume over total volume. Pore structures like pore size, morphology and distribution of porosity within the pressed part present critical items in the load-bearing sections. The load bearing has a large influence on the mechanical properties [1,2,3]. 

Modern processing for producing PM components with adequate properties is through severe plastic deformation (SPD) [4,5,6,7,8]. Among the different SPD techniques, equal channel angular pressing (ECAP) has proven to be promising, enabling the production of ultra-fine grained (UFG) materials. ECAP can be used for the processing of solid metals, but also for the consolidation of metallic powders [9]. 

ECAP was first developed as a processing route over thirty-years ago [10], and subsequently, it was recognized that it was possible to use this procedure to produce materials [11] that have exceptionally refined microstructures. 

**Figure 1:**
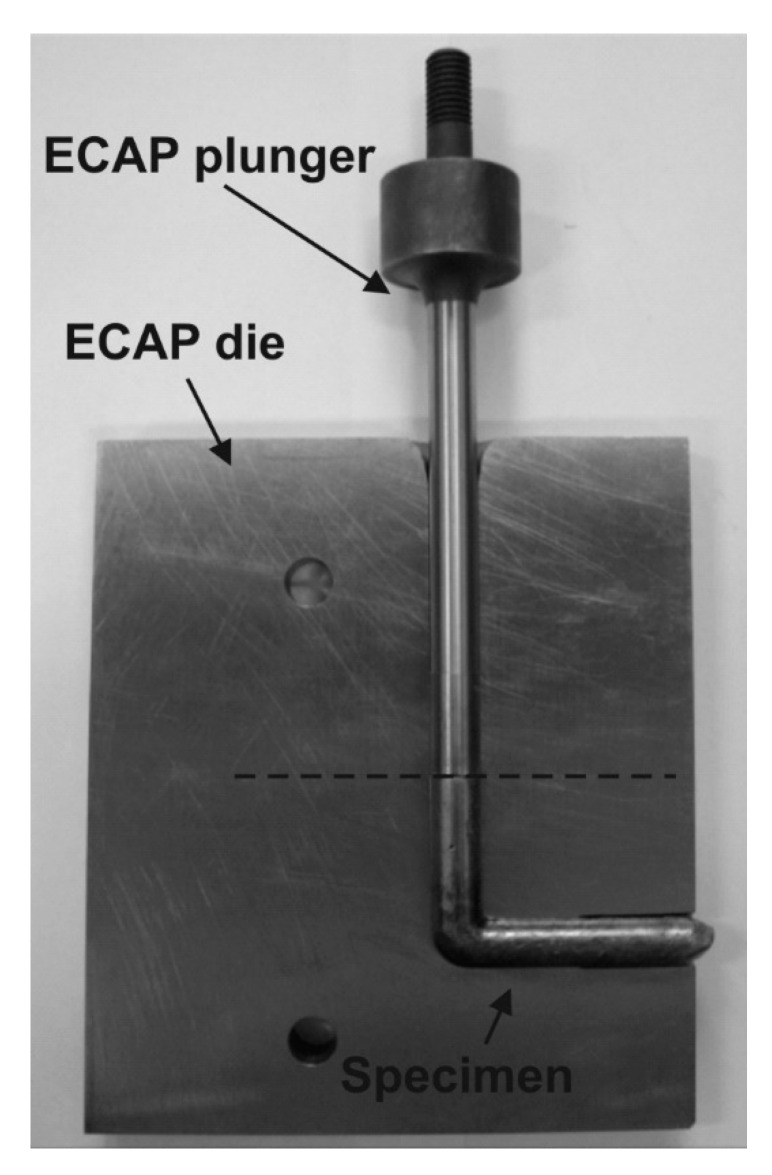
Schema of equal channel angular pressing (ECAP).

During ECAP [9,11,12], the specimen is machined to fit within the channel, and it is then pressed through the die using a plunger, Figure 1. 

Simple shear occurs when the sample passes through the theoretical shear plane, which delineates the plane of intersection between the two separate parts of the channel. Since the cross-sectional dimension of the specimen remains unchanged when it passes through the die, it is feasible to repetitively press the same specimen to impose total strains to attain severe plastic deformation conditions. ECAP can overcome this well-known problem in aluminum alloys with a very stable oxide layer. The severe shear strain, mainly represented by the predominantly compressive stresses, allows breaking of the oxide layers.

On the other hand, in ECAP, some difficulties may arise. Some materials, showing a limited number of slip systems, are difficult to process by ECAP, due to the potential for segmentation of the billet and multiple cracking when pressing at room temperature [13]. Metastable precipitates, limiting the deformability of the billets [14], may be obtained when processing an aluminum-based alloy at room temperature by ECAP. If a high temperature is applied in the process, this may lead to a large-grain-sized microstructure [15].

Iwahashi et al. [16] proposed an analytical expression for calculating the equivalent strain imposed in each ECAP pass in terms of only the die geometric parameters. The maximum equivalent strain for a single ECAP pass is obtained, according to [17] for the die at a 90° angle with sharp corners, ε = 1.15. Therefore, it is important to note that the same strengthening is not attained if a strain of approximately 1 is imposed using alternative forming processing procedures [18]. 

The aforementioned reasons, coupled to the risk of damaging the samples for each further ECAP pass and to the need of reducing the production of scraps to the minimum amount, led to the application of a single ECAP pass for the deformation of the materials, as investigated in the present study. ECAP could eventually be incorporated in the post-processing of Al-alloys obtained through PM. 

This study aimed at quantifying, by means of the standard stereology test methods, the changes in the pore structure of PM materials determined by the application of ECAP to press-and-sinter structures. Pore size dimensions, morphology and distribution of porosity represent critical items in the load-bearing sections and control the final mechanical properties. The final pore shapes of the investigated material visibly changed from particle-like (typical of press-and-sinter structures) to ellipse-like pore (peculiar of the ECAP process). Significant improvements in material properties can, therefore, be obtained by means of SPDs, coupled to an increase of information deriving from the evaluation and study of microstructural dis-homogeneities such as pores. Strain-induced porosity may, however, limit the enhancements achieved in these properties.

## 2. Materials and Methods 

A commercial ready-to-press aluminum-based powder ECKA Alumix 321 (ECKA Granules Germany GmbH, Velden, Germany) (Al-0.95Mg-0.49Si-0.21Cu-0.07Fe-1.5lub) was used as the material to be investigated. 

The pressing of the specimens was carried out by a hydraulic press (2000 kN). The impact energy samples (unnotched) of 55 × 10 × 10 mm were obtained [19] at two different pressing pressures from 400 to 700 MPa. Specimens were dewaxed in a ventilated furnace type of Nabertherm (Nabertherm GmbH, Lilienthal, Germany) at 400 °C for 3600 s. Sintering was performed in a vacuum furnace type of TAV (TAV vacuum furnace, Caravaggio, Italy) at 610 °C for 1800 s, with a cooling rate of 6 °C/s. Density (porosity is a reciprocal value) was measured using the Archimedes technique. Microhardness was carried out by means of Duramin-5 Tester (Struers GmbH, Roztoky u Prahy, Czech Republic) on a minimum of 15 points.

The ECAP of the as-sintered samples was realized by hydraulic equipment (the maximum force of 1 MN) at room temperature. The die channel angle was 90°. Channels in the cross-section have a diameter of 10 mm. The specimens were ECAPed for one pass. 

The samples for microstructural evaluation were examined conventionally by cross-sectional observation methods. The microstructural characterization, as well as fracture investigation, was carried out on un-etched specimens using an optical microscope (OM) LEICA MPEF4 (Leica microsystems, Buccinasco, Italy) equipped with an image analyser and scanning electron microscope (SEM) Jeol 7000F (Jeol, Welwyn Garden City, England). Characterization was carried out at 100 × on a minimum of 5 different image fields for samples prepared by press-and-sinter and 500 × for ECAPed samples. In this way, pores were recorded and processed by a Leica Qwin image analysis system, also for the determination of porosity contents. Quantitative image analysis of the investigated material treats pores as isolated plane two-dimensional objects in the surrounding solid. Regarding the analyses of the dimensional and morphological characteristics of porosity, D_circle_ (representing the diameter of the equivalent circle showing the same area as the metallographic cross-section of the pore) and aspect ratio A (representing the ratio between major axis and minor axis of ellipse equivalent to pore; the aspect ratio considers the stress and strain situation in the workpiece during ECAP), as well as the morphological characteristics f_shape_ and f_circle_. The description of the parameters are as follows [20,21]:

D_circle_, Aspect, and the morphological characteristics f_shape_ and f_circle_ were measured for each pore individually in order to describe the dimensional and morphological characteristics. The calculations of both parameters are reported as follows:

(1) $$f_{shape}\;=\;\frac{D_{\min}}{D_{\max}}=\frac{a}{b}\;/-$$

where D_min_ (cm), the parameter representing the minimum Feret diameter; D_max_ (cm), the parameter representing the maximum Feret diameter; and

(2) $$f_{circle}\;=\;\frac{4\cdot \pi \cdot A}{P^{2}}\;/-$$

where A (cm^2^), the area of the metallographic cross-section of the pore,

(3) A = π⋅a⋅b 

P (cm), the perimeter of the metallographic cross-section of the pore,

(4) $$P=\;\pi \left[1.5\cdot \left(a\cdot b\right)-\sqrt{a\cdot b}\right]$$

The size and the internal notch effect of the pores from the mechanical fracture point of view, represent a decisive point for the material performance. Small rounded nanopores with a lower notch effect are less harmful for the final mechanical behavior. For this reason, only pores with sizes bigger than 100 nm were investigated as potential fracture initiation sites. Hence, all smaller nanopores were excluded from further investigation. 

Some conditions were selected for tensile testing. Specimen dimensions for sinter-and-press specimens were “dog bone” dimension according to the MPIF standard 10 [22] and for ECAPed specimens dimensions that were 10 mm in diameter and 55 mm in length at the thinner gauge section were tested in a universal servo-hydraulic testing machine Tinius Olsen (Tinius Olsen Ltd., Salfords, England) with a crosshead speed of 0.5 mm.min-1. Five measurements were taken from each sample and the average value of three measurements, excluding the highest and lowest values, was recorded. 

## 3. Results and Discussion

### 3.1. Image Analysis

The quantitative stereology established the relationship between measured elements in 3D space and observed profiles on a 2D cross-section plane that randomly intersects these elements [23]. These methods have been used in various fields to obtain the spatial size distribution of measured points from measurement on a random test. For porosity and pore size evaluation, two-dimensional (2D) quantitative image analysis of Optical Microscope (OM) and Scanning Electron Microscope (SEM) images are used, either on polished surfaces or on fresh fracture surfaces. The stereological analysis of the pore structure provides additional information about the average size of the pores, the shape of the pore, and the total area of porosity. On the other hand, it does not provide any topological information about the possible pores interconnection but, as underlined in [24], this is a fast, non-destructive approach to determine the main 3D pore population characteristics from 2D data. The direct determination based on 3D reconstruction technologies from stepwise sectioning (e.g., by using focus ion beam or surface polishing) is much more time-consuming and it is destructive for the sample.

Image analysis is, according to Vander Voort [25,26,27], a process of data transformation in which the initial data set is an image or a collection of images, but the final, resulting data set has another format. So, image processing is just a part of the image analysis process. Practical experience shows that the vast majority of problems in using image analysis stem from inadequate specimen preparation and choosing the wrong etching technique to reveal the structure. Once a decent image is captured, the rest is relatively simple.

The results of the collection the 2D data from quantitative image analysis of the pore structure in aluminum alloys are presented in Table 1.

**Figure 2:**
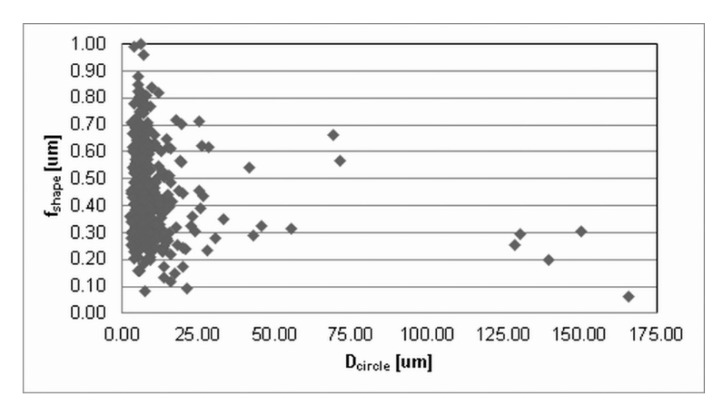
The dimensional and morphological characteristics, f_shape_, of porosity for press-and-sinter specimens (400 MPa).

**Figure 3:**
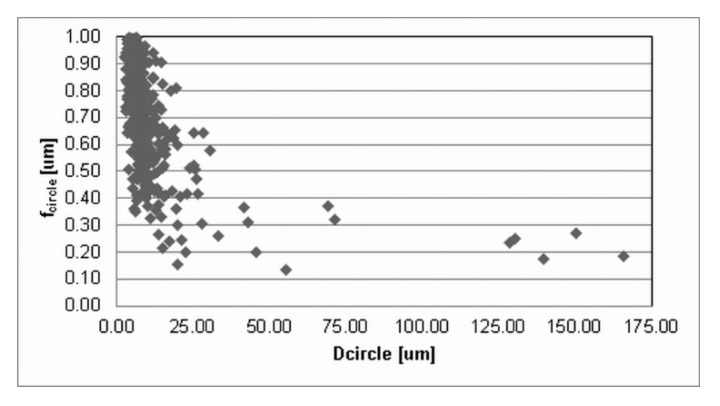
The dimensional and morphological characteristics, f_circle_, of porosity for press-and-sinter specimens (400 MPa).

The press-and-sinter values of particle-like pores’ size D_circle_ are 30.64 μm and 21.27 μm for 400 MPa and 700 MPa, respectively. The contact area between the particles still increases, and the cusp-shaped pores provided the final dimension of pores in the approximate range 10 μm (Figure 2 and Figure 3, examples of pore structure variety for applied pressing pressure of 400 MPa).

### 3.2. Processing of Aluminium PM Alloy

Creation and development of pores during the various processing stages in the PM material reduces the mechanical properties. Press-and-sinter is a conventional PM forming process used for the production of several different components, usually with low processing costs. It produces precisely finished parts, with close to the die dimensions, but also characterized by the presence of porosity at around 10 %, thus with generally lower mechanical properties than wrought products.

**Figure 4:**
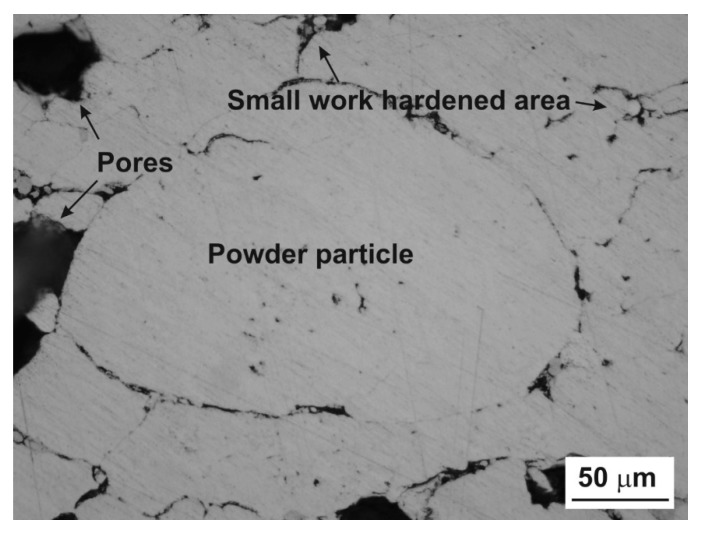
Typical microstructural overview for low pressure.

**Figure 5:**
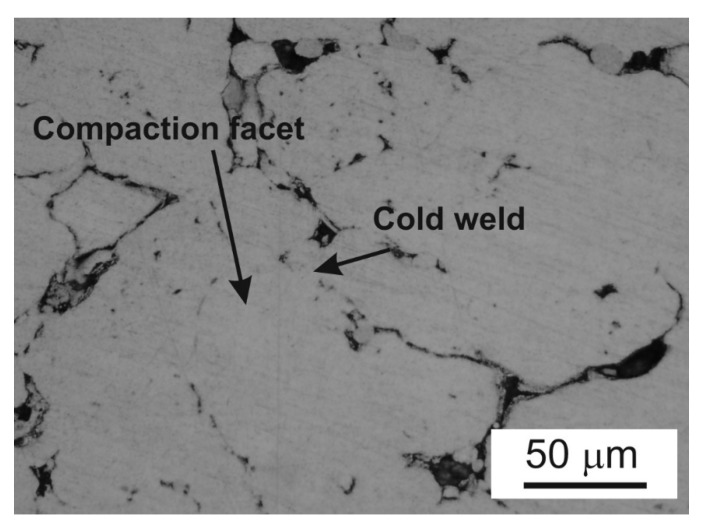
Desirable cold welding in investigated aluminum powder metallurgy (PM) alloy.

During the compaction phase, particularly for low pressures, the densification of the powder occurs by translations and rotations of powder particles, which create a high volume of porosity. Low applied compacting pressures lead to a low green strength. When the particle rearrangement ends, the elastic and plastic deformations of powder particles start through their compaction facets (Figure 4 and Figure 5). 

**Figure 6:**
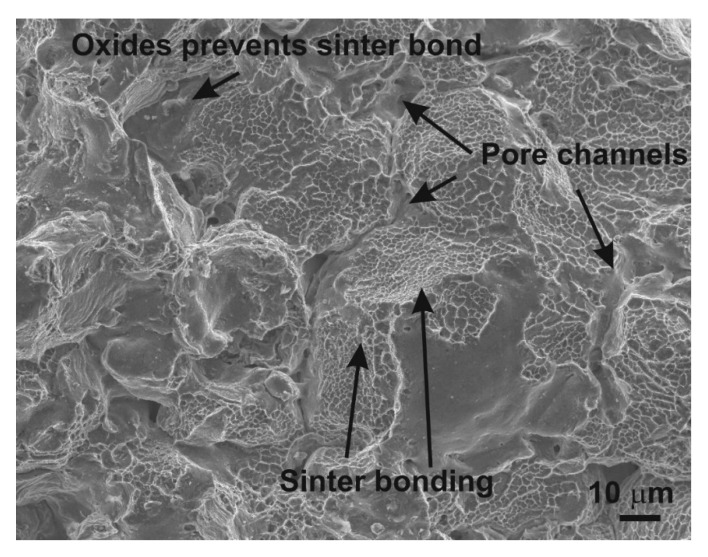
Sintering process and their typical features.

These potential areas for nucleation and growth of inter-particle necks or cold welding during the sintering are increased. A second and important step in PM processing is sintering. During the thermal processing, the cold welds progressively evolve to sintering necks, by means of diffusion-based mechanisms, Figure 6.

**Figure 7:**
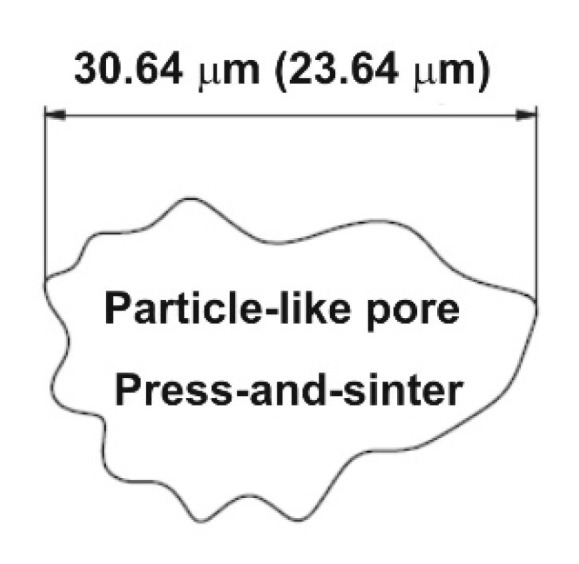
Particle-like pore derived from press-and-sinter process.

The necks have been considered as the load-bearing sections and pores play a role such as micro notches [28]. As for the sintering of aluminum alloys, it results in the formation of secondary porosity during transient liquid-phase sintering (LPS), which is dependent on the previous formation of a liquid able to migrate away from the site of the prior alloying particles [29,30]. Therefore, the morphological and dimensional characteristic results show that the material contains particle-like pore shapes (Figure 7). 

**Figure 8:**
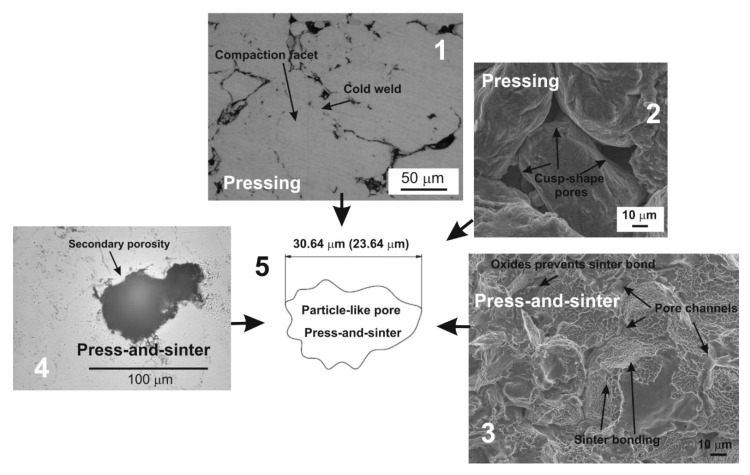
Overview of the press-and-sinter process in aluminum PM alloy.

The values of particle-like pores’ size D_circle_ are 30.64 μm and 23.64 μm for 400 MPa and 600 MPa, respectively. The compressibility results show that the pressing pressure of 400 MPa seems to be appropriate for achieving the desirable cold welding (Figure 8, point 1). Compressibility behavior is reported in papers [21,31]. The final stages of densification are reached, applying the pressure of 600 MPa. The contact area between the particles still increases, and the final stage of compaction produced the cusp-shaped pores in the approximate range from 5 to 10 μm (Figure 8, point 2). Observing Figure 6, it is clearly possible to recognize the typical stages of LPS of the investigated aluminum alloys. The alloying particles dissolve at first in the aluminum matrix and then, by means of the formation of the wetting liquid, they migrate through pore channels and/or the grain boundaries. 

Values reported in Table 1 concerning the morphological characteristics of f_shape_ and f_circle_ indicate that there is a similarity for the entire set of applied processing conditions. On the other hand, Figure 2 and Figure 3 clearly show differences in pore structures; however, the stereology statistics used initially were inadequate. Murphy [32] declared that obvious changes in the loss of particle boundaries were characterized and quantified properly, but the smoothing of the pore edges with the resultant loss in a specific area was not detected. It is a well-known phenomenon [5,30,33] that an atomized technique produces most surface oxides on the aluminum base alloys, which are removed in a subsequent annealing process; nevertheless, a layer of oxides will still cover the surfaces of the heat-treated powders. It is important to reduce surface oxides during the sintering process in order to get a proper “sinter neck” between the adjacent particles.

The effects of LPS led to the generation of mainly secondary pores in the approximate range from 23 to 31 μm. The large value of pores’ size D_circle_ causes the coarse additive particle sizes to generate large residual pores, some of which have a dimension of about 100 μm (Figure 8, 4). A mix of primary, secondary and residual porosity then co-exist in the material. The increase of the pressing pressure resulted in a decrease of the pores’ size D_circle_. The relative oxide/aluminum diffusion rates contribute to the generation of stable oxides on the particle surface which prevent suitable sinter necks; the mechanical properties are 151 MPa and 162 MPa for 400 MPa and 600 MPa, respectively. Balog et al. [5] show the disruption of oxide on pure aluminum powder into oxide nanoscale particles (50–150 nm) along the elongated aluminum particle boundaries by one pass ECAP. It means that, in order to achieve a good sinter neck, aluminum alloys have to be modified by additional steps such as, proper plastic deformation (ECAP, ECAR, direct extrusion) or, in terms of pre-processing, master alloy powders or pre-alloyed powders [5,29,30,34]. 

In terms of the final achieved mechanical properties, several studies focused on the strengthening of aluminum alloys using single and multiple ECAP passes [2,8,9,21]. The interaction of severe shear due to ECAP and surface oxides, which are unbreakable both during the severe deformation and in the previous press-and-sinter, are, therefore, present in the component. The high levels of both compressive and tensile stresses produced in the specimen during the ECAP process, combined with the high localized shear strains, may be sufficient to promote high rates of pore reduction and nucleation. The applied mean stress induces new plasticity-driven densification mechanisms, as well as a stress-assisted diffusion mechanism [35]. The grain refinement during ECAP is an increase in the density of triple junctions that produce strain-induced porosity. This can act as preferred sites for nanopore nucleation [4,21,36,37].

**Figure 9:**
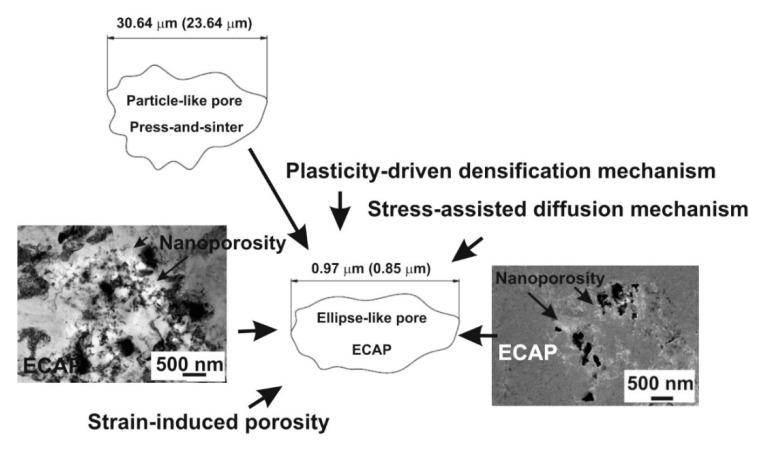
Particle-like pore derived from press-and-sinter process.

Therefore, ECAP induces nanoporosity (Figure 9) in the structure as a process that releases high local stresses created by lattice dislocation pile-ups that stop at grain boundaries, or grain boundary dislocation pile-ups that stop at triple junctions and ledges of grain boundaries [38]. ECAP decreases the porosity content and it can also align particles. The dimensional parameters D_circle_ and Aspect decreases for both conditions from 30.64 to 0.97 μm and from 23.64 to 0.85 μm; and from 2.24 to 1.88 and from 2.21 to 1.82, in the case of pressing at 400 MPa and 600 MPa, respectively. Therefore, the morphological and dimensional characteristic results show that the material contains ellipse-like pore shapes (Figure 9). The main positive effect of ECAP related to the mechanical properties is the suitable deformation welding between the powder particles, the increase in the mechanical properties are from 151 MPa to 203 MPa and from 162 MPa to 221 MPa, in the case of pressing at 400 MPa and 600 MPa, respectively. The deformation neck may be increased by particles deformation under local constraints during the ECAP. They subsequently increase the interfacial area and, therefore, create stronger bonding and stability between the particles. ECAP could, therefore, be seen as a potential strengthening mechanism for achieving the desirable mechanical properties.

**Figure 10:**
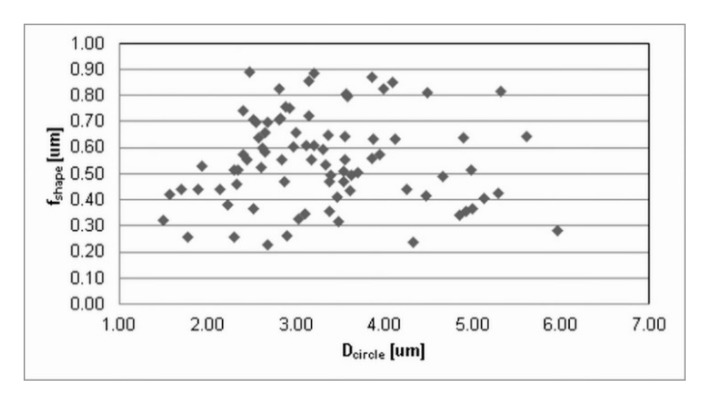
The dimensional and morphological characteristics, f_shape_, of porosity for ECAPed specimens (400 MPa).

**Figure 11:**
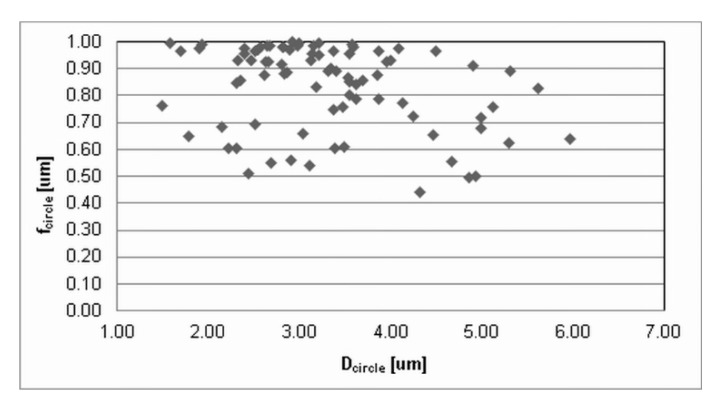
The dimensional and morphological characteristics, f_circle_, of porosity for ECAPed specimens (400 MPa).

Values reported in Table 1 and images in Figure 10 and Figure 11 indicate that the ECAP values of ellipse-like pores D_circle_, due to the aligning effects on particles, are lower than 1 μm: 0.97 μm and 0.79 μm for 400 MPa and 700 MPa, respectively.

ECAP influences the morphological characteristics f_shape_ and f_circle_. This is mainly due to the fact that pores extend primarily in one direction, due to the stress–strain situation during the Severe Plastic Deformation [39,40,41]. Pores became nano sized and more closed and isolated. Nevertheless, strain during the ECAP process determines the formation of induced nanoporosity. 

Compaction during PM production dictates the stress and density distribution in the green compact prior to sintering. The applied process parameters have a strong influence on the overall strength. For low pressures, the densification of the powder occurs by translations and rotations of powder particles, which create a high volume of porosity. When the particle rearrangement is ending, the elastic and plastic deformation of powder particles begin through their compaction facets. Moreover, they allow for the quantification of the intensity of the development of compaction facets. The dimensions of particle contact areas depend primarily on particle shape and the localization of plastic deformation depends on surface geometry and pressure level. This means that the compaction facets, as the result of the overall compressibility effect, depend on the granulometry, compaction pressure, and particle surface roughness form discontinuous adhesive and mechanical particle contacts. Geometrical rearrangement, along with plastic deformation, plays an important role during the densification process. These geometrical characteristics make compaction facets as potential areas for nucleation and growth of inter-particle necks or cold welding during the sintering. Pore size dimensions, morphology and distribution of porosity are critical factors in the load-bearing sections and control the final mechanical properties. Final pore shapes of the investigated material visibly changed from particle-like (press-and-sinter process) to ellipse-like pores (ECAP process). 

## 4. Conclusions

This study aimed at quantifying, by means of the standard stereology test methods, changes in the pore structure of PM materials, determined by the application of ECAP processes to press-and-sinter structures. Pore size, morphology and distribution of porosity within the pressed part represent critical items in the load-bearing sections that represent the controlling mechanism of the mechanical response. Pore shapes can be tailored from particle-like to ellipse-like pores for the press-and-sinter process to ECAPed process, respectively. Hence, the final pore structure parameters depend on the applied plastic deformation. 

Significant improvements in material properties can, therefore, be obtained by means of the SPD, accompanied with knowledge improvements in microstructural inhomogeneities such as porosity. However, strain-induced porosity may limit the enhancements that can be achieved in these properties.

## Figures and Tables

**Table 1:** The dimensional and morphological characteristics of porosity, as well as porosity value and tensile strength for investigated aluminum alloy.

Applied Process	Pressing Pressure	f_circle_	f_shape_	D_circle_	Aspect	P*	Rm
	[MPa]	[-]	[-]	[µm]	[µm]	[%]	[MPa]
press-and-sinter	400	0.92	0.70	30.64	2.24	7.88	151
ecap		0.91	0.67	0.97	1.88	1.69	203
press-and-sinter	500	0.93	0.72	30.20	nd**	7.60	162
ecap		0.91	0.65	0.90	nd	1.61	215
press-and-sinter	600	0.92	0.69	23.64	2.21	7.18	162
ecap		0.91	0.67	0.85	1.82	1.42	221
press-and-sinter		0.89	0.64	21.27	nd	6.91	164
ecap	700	0.9	0.64	0.79	nd	1.36	234

* P – Porosity, ** not determined.
